# Uganda’s experience in establishing an electronic compendium for public health emergencies

**DOI:** 10.1371/journal.pgph.0001402

**Published:** 2023-02-10

**Authors:** Alex Riolexus Ario, Dativa M. Aliddeki, Daniel Kadobera, Lilian Bulage, Joshua Kayiwa, Milton M. Wetaka, Simon Kyazze, Felix Ocom, Issa Makumbi, Paul Mbaka, Prosper Behumbiize, Immaculate Ayebazibwe, Stephen K. Balinandi, Julius J. Lutwama, Adam Crawley, Nomita Divi, John R. Lule, Joseph C. Ojwang, Julie R. Harris, Amy L. Boore, Lisa J. Nelson, Jeff Borchert, Dennis Jarvis

**Affiliations:** 1 Uganda Public Health Fellowship Program, Kampala, Uganda; 2 Uganda National Institute of Public Health, Kampala, Uganda; 3 Uganda Public Health Emergency Operations Center, Kampala, Uganda; 4 Division of Health Information, Ministry of Health, Kampala, Uganda; 5 Health Information Systems Program, Kampala, Uganda; 6 Uganda Virus Research Institute, Entebbe, Uganda; 7 Ending Pandemics, San Francisco, California, United States of America; 8 US Centers for Disease Control and Prevention, Kampala, Uganda; 9 Division of Vector-Borne Diseases, National Center for Emerging, Zoonotic, Infectious Diseases, Centers for Disease Control and Prevention, Fort Collins, Colorado, United States of America; 10 Division of Global Health Protection, Center for Global Health, Centers for Disease Control and Prevention, Atlanta, Georgia, United States of America; Georgetown University, UNITED STATES

## Abstract

Uganda has implemented several interventions that have contributed to prevention, early detection, and effective response to Public Health Emergencies (PHEs). However, there are gaps in collecting and documenting data on the overall response to these PHEs. We set out to establish a comprehensive electronic database of PHEs that occurred in Uganda since 2000. We constituted a core development team, developed a data dictionary, and worked with Health Information Systems Program (HISP)-Uganda to develop and customize a compendium of PHEs using the electronic Integrated Disease Surveillance and Response (eIDSR) module on the District Health Information Software version 2 (DHIS2) platform. We reviewed literature for retrospective data on PHEs for the compendium. Working with the Uganda Public Health Emergency Operations Center (PHEOC), we prospectively updated the compendium with real-time data on reported PHEs. We developed a user’s guide to support future data entry teams. An operational compendium was developed within the eIDSR module of the DHIS2 platform. The variables for PHEs data collection include those that identify the type, location, nature and time to response of each PHE. The compendium has been updated with retrospective PHE data and real-time prospective data collection is ongoing. Data within this compendium is being used to generate information that can guide future outbreak response and management. The compendium development highlights the importance of documenting outbreak detection and response data in a central location for future reference. This data provides an opportunity to evaluate and inform improvements in PHEs response.

## Background

In Uganda, as in most sub-Saharan African countries, communicable diseases still account for most public health emergencies (PHEs) [1]. Uganda is one of the hotspots for emerging and re-emerging infectious disease threats in Eastern Africa [2]. The intrusion of humans into ecological areas formerly occupied by animals and other pathogen carriers such as bats and birds, has resulted in an increased number of disease outbreaks over the last two decades [3–5]. Increasing population size and urbanization has further contributed to the country’s vulnerability to environmental degradation-related emergencies, including floods, landslides, and mudslides, among others [6].

To improve preparedness and response to PHEs, Uganda’s Ministry of Health (MoH) established the Uganda National Institute of Public Health (UNIPH) in 2013. The initial purpose of the UNIPH was to implement core capacities specified in the International Health Regulations 2005 (IHR 2005). These included reinforcing nationwide laboratory systems, establishing a Public Health Emergency Operations Center (PHEOC), functionalizing the Uganda Public Health Fellowship Program (UPHFP) to strengthen workforce development, and enhancing real-time information systems for epidemic preparedness, prevention, detection, and response to PHEs [7]. These interventions have contributed greatly towards improving preparedness and response to PHEs, with improvements in early detection, laboratory turnaround time for diagnostics, and effective coordination of response efforts to outbreaks [8–11].

Despite this progress, there were gaps in documentation of the response efforts to these PHEs, such as data about the timeliness of outbreak detection and response [12–14]. Having a log of historical data can improve preparedness to respond to PHEs as well as provide a benchmark for forecasting future PHEs [15,16]. An electronic database for all PHEs that includes complete timeliness data alongside other contextual information can provide valuable insights into the effectiveness of current surveillance systems, inform necessary modifications or interventions, and apprise the value of rapid response [17]. We established a comprehensive electronic database for PHEs in Uganda since 2000.

## Materials and methods

### Ethics statement

This is an initiative of the Uganda National Institute of Public Health, of which the Uganda Public Health Fellowship Program and the Uganda Public Health Emergency Operations Center are part of. Henceforth, the authors have been granted permission to access data and publish any works that are befitting to share globally. The authority to establish the outbreaks compendium and publish data emanating from it was granted through the Office of the Director General Health Services, Ministry of Health.

### Development team

We constituted a core team to support the compendium development and ensure consensus at each step of the process. Members of the development team were selected from departments and institutions that lead and support outbreak response in Uganda. The team included technical experts (epidemiologists, programmers, and informaticians) from the UPHFP [18], PHEOC [19], and Department of Integrated Epidemiology, Surveillance and Public Health Emergencies (IES&PHE) [20] in MoH, as well as Health Information Systems Program, Uganda (HISP-Uganda) [21], Ending Pandemics [22], and the US Centers for Disease Control and Prevention (CDC) [23]. The development team met physically and virtually on a regular basis to develop content and assess the progress of the compendium development, and provide technical guidance as required.

### Defining Public Health Emergencies (PHEs)

The compendium development core team agreed on events that would be considered as PHEs, focusing on the One Health approach. We adopted the WHO definition of s a PHE as any event caused by an epidemic or pandemic disease, a highly infectious agent, bioterrorism, and natural or artificial disasters that pose substantial health consequences and has the potential to overwhelm the public health or health care system of Uganda. The team resolved to collect data on human, animal, and zoonotic disease outbreaks as well as disasters reported in the country [24,25].

### Development of a data dictionary

We developed a data dictionary of the response milestones to be collected for each PHE. We built on the initial eleven milestones recommended by the 2018 and 2019 International Expert Consultations coordinated by Ending Pandemics, a San Francisco based non-profit organization that provides technical expertise and catalytic funding to partners to improve early detection and rapid response to stop the spread of disease outbreaks [26]. These milestones were refined to ensure applicability across the One Health continuum, the interrelationship among humans, animals, and the environment. We further included milestones relevant to understanding trends in occurrence of PHEs and evaluating the effectiveness of Uganda’s preparedness and response to PHEs over time.

The dictionary provided the names and definitions of the milestones we targeted to collect for each PHE. The data dictionary ensured that we collected quality, standard, and consistent data for all PHEs reported in the country. The selected milestones captured information on the duration, magnitude, temporality, spatiality, fatalities due to the PHE, and the PHE preparedness and response interventions (Table 1).

**Table 1:** Data dictionary of variables included in the compendium.

Variable name	Variable Definition
Emergency	Identify the type of Emergency (Disaster/Disease Outbreak) We defined outbreak as “sudden occurrence of something unwelcome”. For disease conditions, we use the case definitions in the National Integrated Disease Surveillance and Response Guidelines. For an outbreak to be entered into the compendium, means the alert was reported to the Emergency Operations Centre, verified and qualified as an outbreak according to the case definition
Disease classification	Provide a classification for the Disease (Animal/Human/Zoonotic)
Emergency name	Name of emergency
Region	Region of occurrence
District	District of occurrence
Sub-county	Sub-county of occurrence
Parish	Parish of occurrence
Village	Village of occurrence
Predict date	Date occurrence of the PHE was predicted or date an alert of a potential PHE occurrence is availed/given
Prevent date	Date initial preventive interventions were put implemented
Start date	Date of symptom onset of index case or if not available; date of first interface with the health system
Detect date	Earliest date of interface with health system
Verification date	Earliest date of outbreak verification through a reliable mechanism
Confirmation type	How was the outbreak confirmed
Sample/specimen type collected	What was the type of specimen collected
Sample/specimen collection date	Date of first confirmatory sample collection
Sample Transportation Date	Date of specimen transportation to laboratory
Date of sample receipt at Laboratory	Date the sample was received at the laboratory
Laboratory confirmation date	Laboratory confirmation date
Strain	Causative agent strain
Date of MoH Notification	Date of notification to the MoH
Response date	Date and time of initial PH response
Response level	What is the level of the outbreak or emergency response
PHEOC activation	Was EOC activated
PHEOC activation date	Date and time of activation
Level of EOC activation	Level of PHEOC activation
UPHFP engagement date	Date of the UPHFP engagement
Disaster presence	Were there any natural or artificial disasters associated with the outbreak or emergency (war, floods, landslides, earthquake, refugee crisis)
Disaster name	If yes, please indicate the natural or man-made disaster
Season of occurrence	Division of the year marked by dryness or wetness during which the outbreak or emergency started
Animal sector outbreak presence	Was there another disease outbreak or emergency among animals at the time of the disease outbreak
Outbreak name	If yes, please indicate the outbreak or emergency
Transboundary outbreak	Did the outbreak cross borders
Cross-border country	If yes, which country
Transboundary response	Was there cross border collaboration in the response
Suspect cases count	Total suspect case count
Probable case count	Total probable case count
Confirmed case count	Total confirmed case count
Fatalities	All deaths (homicide, suicide, accident, natural); Total fatality count, Cause specific counts and Excess deaths
Health worker infections	Number of affected health workers
Declaration date	Date of the outbreak declaration
WHO notification date	What was WHO outbreak notification date
Ongoing PHE	Is this outbreak still ongoing
PHE end date	If no, date of end of outbreak declaration
AAR conducted	Was an After-Action Review completed for the PHE?
Date AAR conducted	Date when the AAR was conducted

### Configuration of the compendium

We worked with HISP Uganda to design an online data entry form, using the milestones listed in the dictionary. The online data entry form was designed under the electronic Integrated Disease Surveillance and Response (eIDSR) system currently on the DHIS2 platform. The eIDSR is an online tool designed to enable real-time reporting of priority IDSR diseases or conditions that constitute PHEs. DHIS2 is a web-based open-source platform for the collection, validation, analysis, and reporting of national health data; and is interoperable with other systems, both internal and external, which enables easy sharing of information.

### Time period covered by the compendium

We agreed to construct a compendium covering disease outbreaks that occurred over a period of 20 years, beginning in 2000. We were cognizant of the data gaps which existed in the earlier years of the Epidemiology and Surveillance Division of the MoH prior to the establishment of the PHEOC.

### Data collection

We worked with the UPHFP advanced field epidemiology fellows to collect both retrospective and prospective (as outbreaks occurred and alerts were received) data on PHEs in Uganda since 2000. The retrospective data collection process involved identification and collaboration with relevant sectors and agencies of government that play a key role in preparedness and response to PHEs in Uganda, to seek access to any data related to PHEs that occurred in Uganda since 2000. In addition, we reviewed literature from multiple sources to identify information on retrospective PHEs reported in Uganda. The sources of literature included epidemiological investigation reports and publications, laboratory records, district weekly, and monthly reports, and correspondences between key PHE subject matter experts. Furthermore, we reviewed seasonal data from the National Emergency Coordination and Operations Center (NECOC) of the directorate of disaster management in the Office of the Prime Minister (OPM) for data on any human associated disasters such as floods, landslides, armed conflicts, refugee crises, insect/pest, and animal infestations, famine and prolonged dry weather spells among others. We also reviewed event-based surveillance data from print and electronic media for any information on reported past and ongoing PHEs.

**Fig 1:**
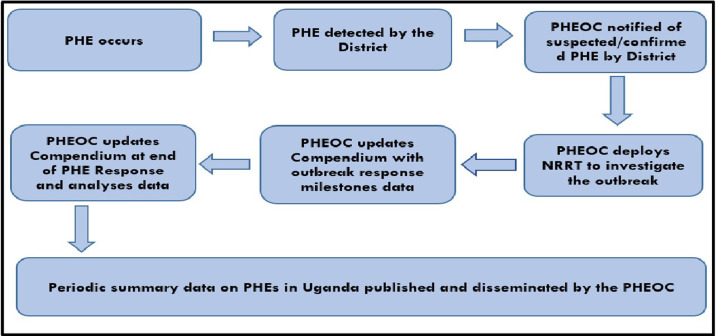
Process of prospective data collection and entry into the compendium.

We compiled the data collected from all the above sources into a MS Excel sheet. We further reviewed and verified this data for quality (accuracy, completeness, reliability, relevance, and timeliness), before starting the data entry process. The prospective data collection process required the real-time collection of data on PHEs throughout the detection, verification, confirmation, preparedness, and response process (Fig 1).

### Data entry

We entered the data into the data entry form customized in the eIDSR system. The initial data entry process was used to pre-test the compendium. This process enabled the inclusion of omission/commission patterns and quality checks which not only simplified the data entry process but also ensured that quality data is captured into the compendium.

## Results

### The compendium

A national PHE compendium was developed within the eIDSR system. The documented variables for PHEs data collection included: PHE type, location, and outbreak timeline metrics for each PHE. The compendium was updated with retrospective PHEs data and for any occurring or recurring PHE. Prospective data collection was captured in real-time, which boosted the completeness of the data. Retrospective data for PHEs which was published were complete, but others were incomplete. Further analysis of timeliness metrics for PHEs is currently being conducted and will be presented in a subsequent manuscript.

### Distribution of public health emergencies in Uganda, 2000–2020

**Fig 2:**
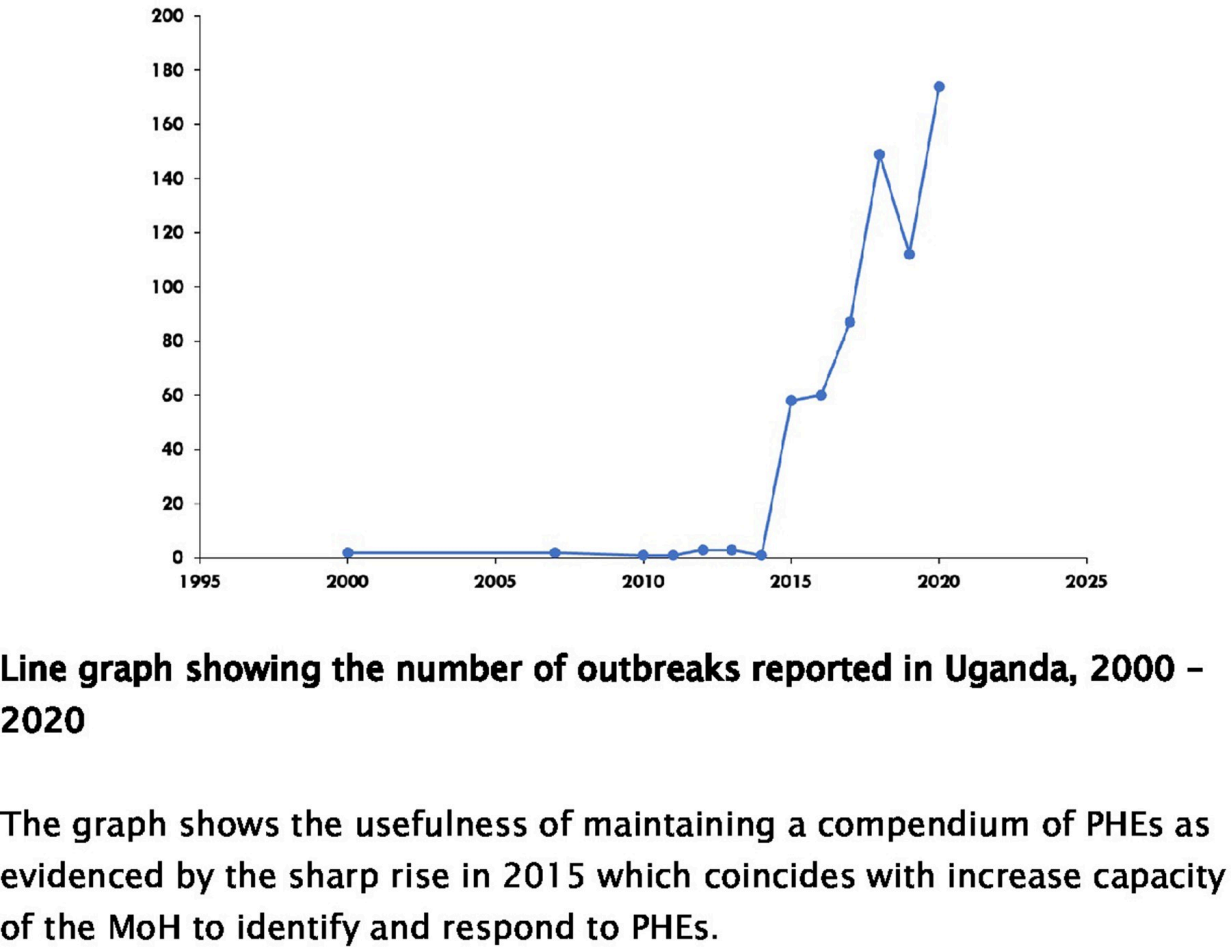
The number of outbreaks reported in Uganda, 2000–2020.

**Fig 3:**
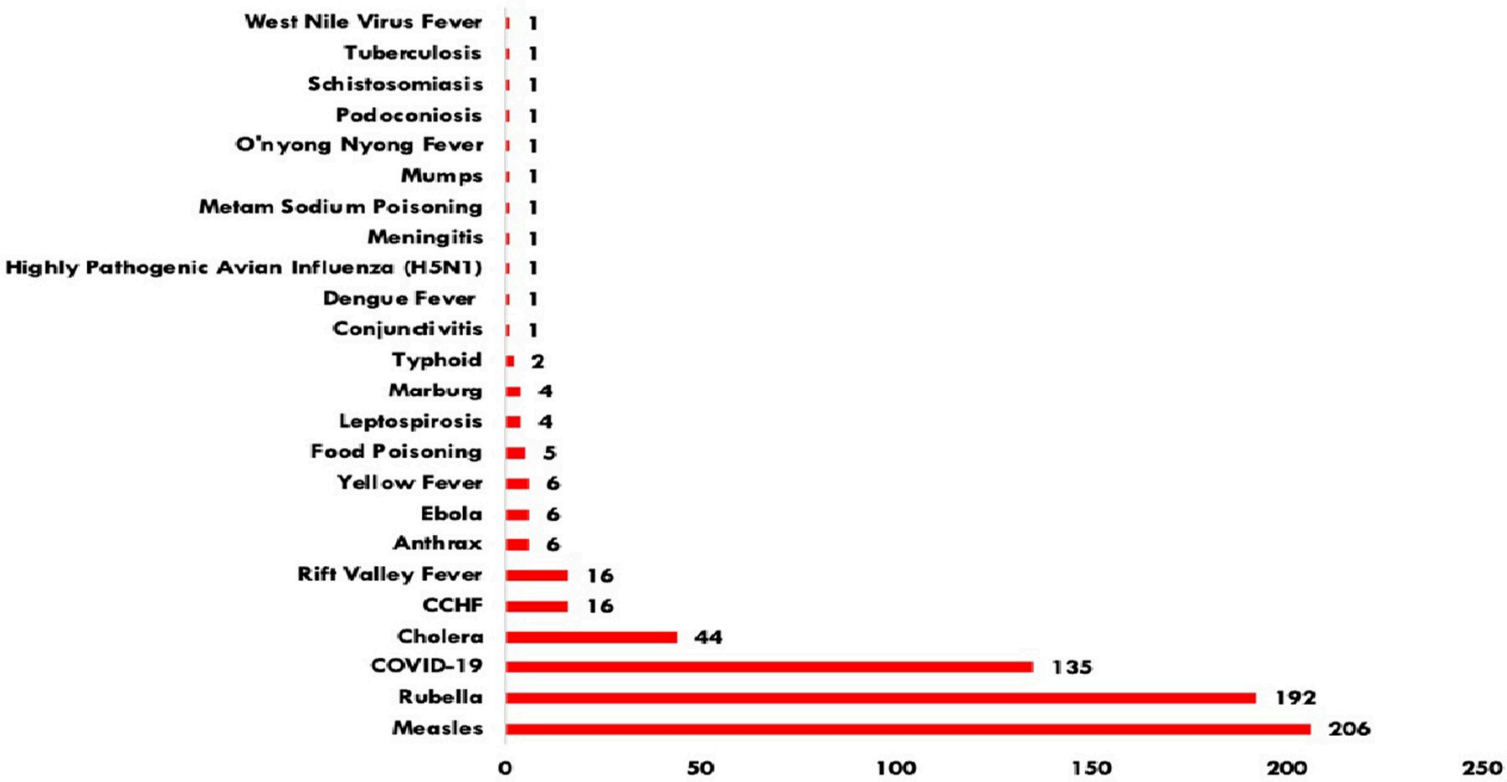
Frequency of reported outbreaks in Uganda, 2000–2020.

Fig 2 below shows the number of outbreaks reported in Uganda from 2000 to 2020. The graph demonstrates the usefulness of maintaining a compendium of PHEs as evidenced by the sharp rise in 2015 which coincides with increased capacity of the MoH to identify and respond to PHEs. By December 2020, the compendium included 833 disease outbreaks, 295 (35%) of which were recorded prospectively. Of these, 435 (52%) represented vaccine-preventable diseases; 180 (22%) were zoonoses (diseases transmitted to humans from animals); and 49 (6%) were specifically Viral Hemorrhagic Fevers (VHFs) (Fig 3).

The most frequent disease outbreaks detected were measles (206/833; 25%), rubella (192/833; 23%), malaria (183/833; 22%) and COVID-19 (133/833; 16%). Malaria outbreaks were reported in 96 (71%) districts during 2019 and 81 (60%) districts in 2020. Most outbreaks were detected in the Northern (27%) and Western (27%) regions of Uganda. Central Uganda reported the fewest outbreaks (22%).

### Distribution of measles outbreaks in Uganda, 2015–2020

**Fig 4:**
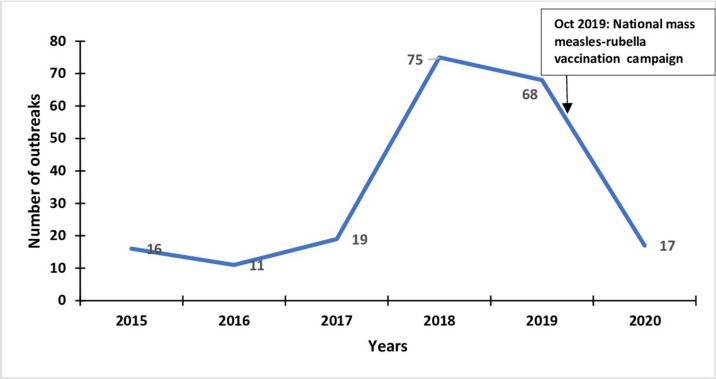
Number of measles outbreaks reported annually in Uganda, 2015–2020.

Between 2015 and 2020, 206 measles outbreaks were registered in the compendium. The highest number of outbreaks was reported in 2018 (75) and 2019 (68). Kamwenge District reported measles outbreaks annually from 2015–2020 (Fig 4). During this period, two national mass measles vaccination campaigns were held. We noticed a slight decline in the number of measles outbreaks following the vaccination campaign in 2015. However, this was followed by an upsurge in 2017, 2018, and early 2019. Following the October 2019 mass measles vaccination campaign, there was a decline in the number of measles outbreaks reported in 2020 (Fig 4).

### Distribution of rubella outbreaks in Uganda, 2015–2020

**Fig 5:**
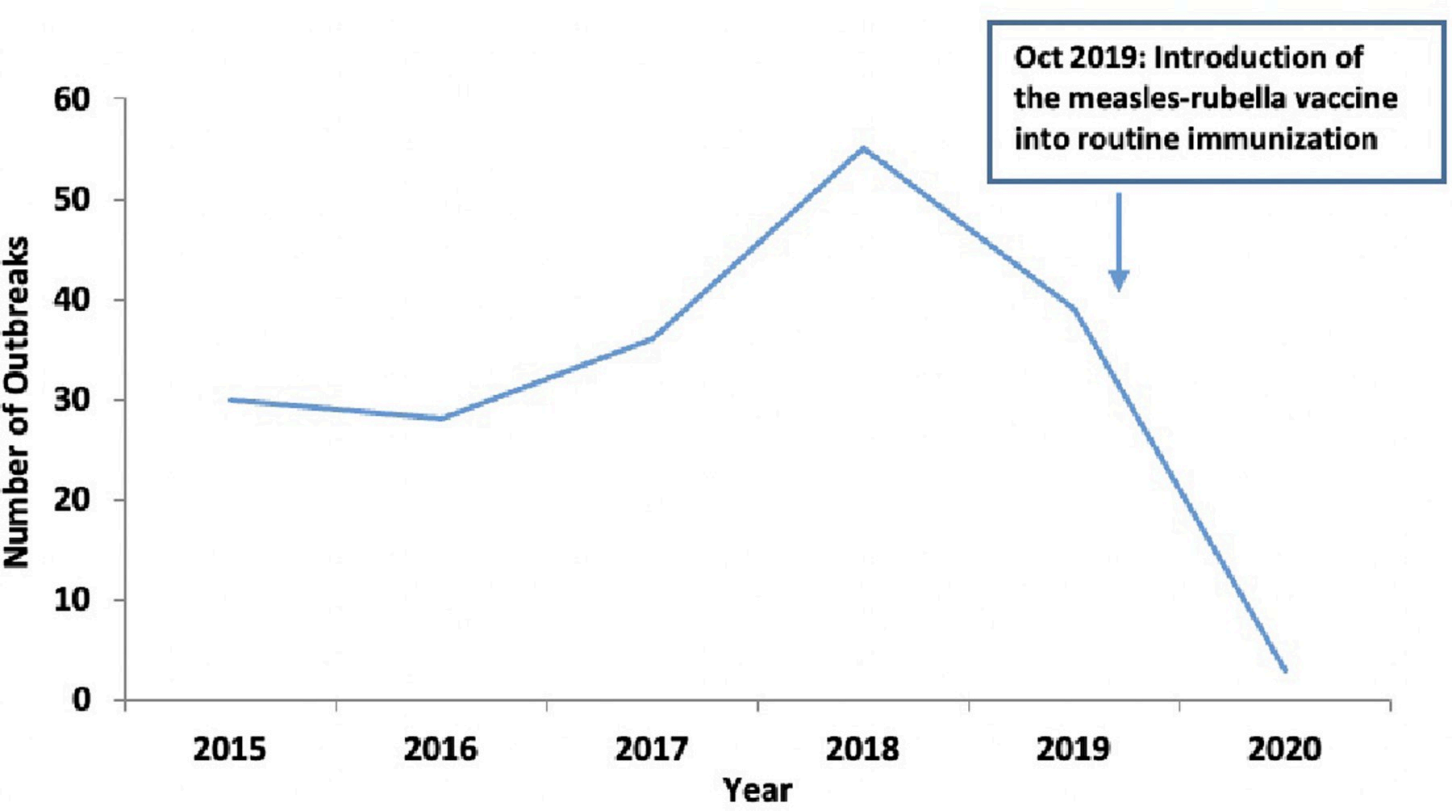
Number of rubella outbreaks reported annually in Uganda, 2015–2020.

Between 2015 and 2020, 192 rubella outbreaks were reported in Uganda, with the highest number of outbreaks occurring in 2018 (55) and 2019 (39) (Fig 5). In October 2019, the measles-rubella vaccine was introduced into the Uganda routine immunization schedule.

### Improvement in the timeliness of detection and response for Ebola and Marburg outbreaks in Uganda, 2000–2020

**Fig 6:**
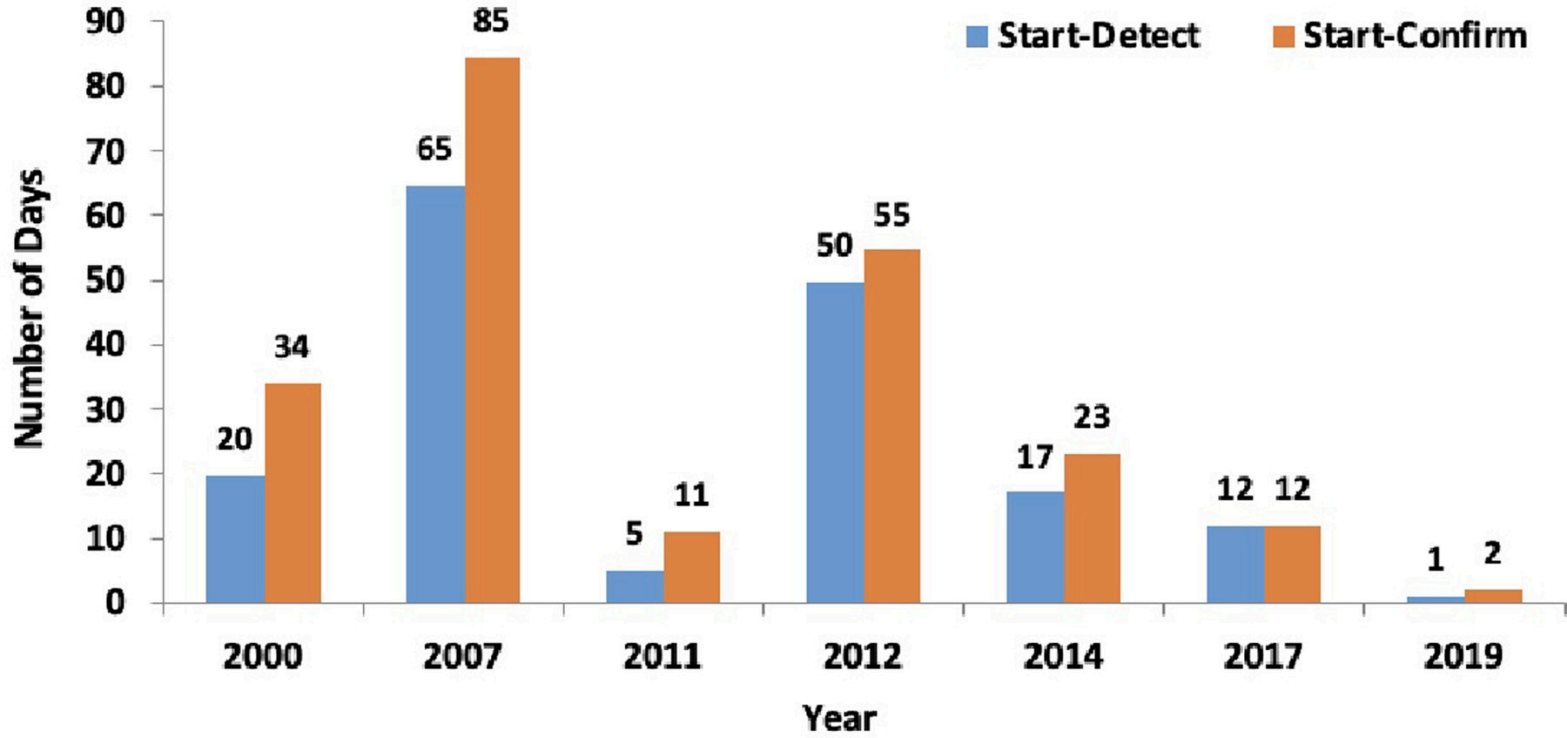
Number of days between the start, detection and confirmation of Ebola and Marburg outbreaks in Uganda, 2000–2020.

Between 2000 and 2020, a total of 12 Ebola and Marburg outbreaks were reported in Uganda. We observed a decline in the number of days from the outbreak start date (date of symptom onset of the index case) to outbreak detection (earliest date of interface with the health system) and to outbreak confirmation (Fig 6).

### Distribution of national disasters in the compendium

For national disasters, we collected data only for the year 2020, and we registered a total of 59 occurrences. The most frequent disasters were floods (47%) and landslides (17%). The northern region reported the most floods (39%), while Central Region had the fewest floods (8%); landslides were reported mainly in the Eastern (Mt. Elgon) and Western (Mt. Rwenzori) regions.

## Discussion

We established a compendium for PHEs in Uganda, through the development of a database of both retrospective and prospective data on PHEs. This activity profiled PHEs that occurred in Uganda, in terms of their duration, magnitude, temporality, spatiality, fatalities, preparedness, and response efforts. Uganda now has a central archive for data and information regarding the sequence of detection and response to PHEs and will continue to collect data on a number of variables that are relevant to outbreak detection and response.

Compendium analysis indicated that non-zoonotic disease outbreaks were the leading contributors to the overall burden of the PHEs in Uganda, followed by zoonotic diseases. Uganda has experienced a steady increase in the number of zoonotic disease outbreaks over the past few years [27]. This finding informs our preparedness and response efforts and calls for the strengthening of the One Health Framework, which brings on board multi-sectoral stakeholders with multidisciplinary response capacities [28].

Additionally, measles and rubella were the diseases that contributed significantly to the burden of disease outbreaks reported between 2015 and 2020. These findings call for conducting periodic supplementary immunization activities to cover the immunity gaps caused by large numbers of children not being reached during routine immunization activities [29–32]. Similarly, based on the country outbreak information, Uganda should consider introducing more vaccines into the routine immunization schedule, as observed by the decline in rubella outbreaks following the introduction of the measles-rubella vaccine into routine immunization [33,34]. The compendium data can also trigger investigations to determine vaccine effectiveness and inform the designing of interventions for improving immunization program in Uganda. The observed reduction in the number of days for outbreak detection and confirmation has been observed in VHFs, and this was attributed to the enhanced viral hemorrhagic fever surveillance program implemented in Uganda [35]. This observation is vital in the design of surveillance systems for similar programs to enhance early detection and response to public health emergencies.

Since surveillance systems are established to inform decision making and public health actions [36], the compendium, which will be updated in real-time provides a platform for periodic analysis and interpretation of data on PHEs occurrence, reporting, response, and control. This information could be used to study and understand disease outbreak patterns and trends, inform interventions and investments, and act as an early warning system to inform the country’s response efforts. In addition, researchers can use the database to access data on epidemics in the country to help generate new information that can be used globally in epidemic control. While there are several surveillance systems offering alerts and news browsing, there are only a few databases around the world where researchers, government health officials, epidemiologists, and public health practitioners can look for historical event data which are updated in real-time [37].

During the 2017 Joint External Evaluation (JEE) assessment of the country’s IHR core capacities, Uganda committed to prioritizing preparedness through instituting a mechanism for regularly updating its hazard or risk profile [38]. The PHEs compendium will benefit this process and act as a reference point for updating the country’s National Multi-Hazard Preparedness and Response Plan. The compendium also aligns with the vision and goals of the IHR 2005, which advocate for the building of robust national public health systems that are able to maintain active surveillance of public health events [39].

Real-time prospective data collection and entry should be prioritized for all PHEs. We found that retrospective data entry often affected data completeness and accuracy. With the exception of PHE responses that had been fully documented and published, data on other PHEs was, in most cases, incomplete. We managed to collect data on disasters for only the year 2020; this may therefore not represent the true number of disasters that occurred in the country over the years.

The database of PHEs in Uganda has contributed towards improved documentation and profiling of the country’s PHEs by both UPHFP and the PHEOC, enhanced preparedness, early detection, and rapid response to epidemics through the application of public health informatics. This will include enhancing existing information systems for tracking emerging and re-emerging epidemics and directing as well as mobilizing and deploying the limited resources for interventions that address the response, laboratory information flows, monitoring medical countermeasures and stockpiles, analyzing, tracking, and coordinating logistics, and improving the use of information by decision-makers at national, sub-national and community levels. This compendium database will further strengthen multi-sectoral coordination by bringing on board relevant One Health stakeholders and working together to improve surveillance, early warning, preparedness, early detection, and rapid response interventions of PHEs, especially at the source.

## Conclusion

The PHEs compendium in Uganda is established. The compendium is now operational, and data within it are being used to generate information that guides the planning, coordination, and management of outbreak prevention, preparedness, and response interventions. It’s also a resource database for historical data for researchers, public health practitioners, and planners in Uganda and around the world. Developing a compendium for PHEs provides a record of PHEs and their response in Uganda which will enable an in-depth understanding of PHEs, and facilitate prediction of their occurrence. In addition, it will build robust coordination and collaboration mechanisms for preparedness and response interventions to address future PHEs, especially at the source, in Uganda. It is envisaged that this national compendium for PHEs will go a long way to not only address compliance to critical IHR capacities and improve health security for Uganda but also ensure coordinated and collaborative limited resources mobilization and deployment. This approach, if well sustained, could be an excellent opportunity for other resource—limited high disease burden countries to emulate and improve global health security.
